# Digital PCR Validates 8q Dosage as Prognostic Tool in Uveal Melanoma

**DOI:** 10.1371/journal.pone.0116371

**Published:** 2015-03-12

**Authors:** Mieke Versluis, Mark J. de Lange, Sake I. van Pelt, Claudia A. L. Ruivenkamp, Wilma G. M. Kroes, Jinfeng Cao, Martine J. Jager, Gre P. M. Luyten, Pieter A. van der Velden

**Affiliations:** 1 Department of Ophthalmology, Leiden University Medical Center, Leiden, The Netherlands; 2 Department of Clinical Genetics, Laboratory for Diagnostic Genome Analysis (LDGA), Leiden University Medical Center, Leiden, The Netherlands; 3 Department of Ophthalmology, The Second Hospital of Jilin University, Changchun, China; Institut Jacques Monod, FRANCE

## Abstract

**Background:**

Uveal melanoma (UM) development and progression is correlated with specific molecular changes. Recurrent mutations in *GNAQ* and *GNA11* initiate UM development while tumour progression is correlated with monosomy of chromosome 3 and gain of chromosome 8q. Hence, molecular analysis of UM is useful for diagnosis and prognosis. The aim of this study is to evaluate the use of digital PCR (dPCR) for molecular analysis of UM.

**Methods:**

A series of 66 UM was analysed with dPCR for three hotspot mutations in *GNAQ/GNA11* with mutation specific probes. The status of chromosomes 3 and 8 were analysed with genomic probes. The results of dPCR analysis were cross-validated with Sanger sequencing, SNP array analysis, and karyotyping.

**Results:**

Using dPCR, we were able to reconstitute the molecular profile of 66 enucleated UM. With digital PCR, *GNAQ/GNA11* mutations were detected in 60 of the 66 UM. Sanger sequencing revealed three rare variants, and, combined, these assays revealed *GNAQ/GNA11* mutations in 95% of UM. Monosomy 3 was present in 43 and chromosome 8 aberrations in 52 of the 66 UM. Survival analysis showed that increasing 8q copy numbers were positively correlated with metastasis risk.

**Conclusion:**

Molecular analysis with dPCR is fast and sensitive. Just like the recurrent genomic aberrations of chromosome 3 and 8, hotspot mutations in *GNAQ* and *GNA11* are effectively detected in heterogeneous samples. Increased sensitivity contributes to the number of mutations and chromosomal aberrations detected. Moreover, quantification of copy number with dPCR validated 8q dosage as a sensitive prognostic tool in UM, of which implementation in disease prediction models will further improve prognostication.

## Introduction

Uveal Melanoma (UM) is a rare intraocular tumour occurring in the European population with a frequency of 7 cases per million [1]. The primary event in UM is either a mutation in the *GNAQ* or the *GNA11* gene, located respectively on chromosome 9q21.2 and 19p13.3. Since the vast majority of UM displays one of these hotspot mutations, UM can be regarded genetically homogeneous [2,3]. The same holds true for UM progression that is characterized by recurrent genetic aberrations. With classical karyotyping, monosomy of chromosome 3 and gain of chromosome 8q have been discovered and shown to be correlated with UM progression [4,5]. Cytogenetic analysis and fluorescent in situ hybridisation furthermore revealed a dosage effect for additional copies of 8q on survival [4,6]. In this model an increased risk of metastases is observed with increasing 8q copy numbers. Monosomy 3 and an aberrant chromosome 8 often occur together and this combination is correlated with a bad prognosis [7]. Based on the frequency of monosomy 3 and chromosome 8 abnormalities, it has been proposed that chromosome 8 abnormalities are secondary to monosomy 3 [8,9]. Monosomy 3 and 8q gain can be applied in the clinic to set an accurate prognosis but classical karyotyping is devious and may fail because it requires *in vitro* culture of UM cells. Hence alternative methods that do not require *in vitro* culture for molecular characterisation have been developed, such as microsatellite analysis (MSA), multiplex ligation-dependent probe amplification (MLPA), single-nucleotide polymorphisms (SNP) and array CGH [8,10–12]. Chromosome 8 aberrations are also incorporated in these assays, although information on 8q copy number dosage is not routinely acquired to stratify patient risk [4,6].

The concept of dPCR was first put forward in the nineties [13]. Using limiting dilutions of DNA template in hundreds to thousands of parallel PCR reactions, PCR was digitalized. Rather than analysing the cumulative signal, as done in quantitative PCR, the number of individual PCR reactions with the desired amplicon provides an absolute quantification of a DNA sample in digital PCR. When the parallel PCRs are analyzed for amplification at different wavelengths, reference gene and target gene can be measured in the same reaction to calculate copy numbers. Alternatively, using WT and mutation specific probes, mutant and WT alleles can be quantified in one test [14,15]. We evaluated the use of the dPCR for *GNAQ/GNA11* mutation analysis as well as for monosomy 3 and chromosome 8 aberrations in a series of 66 UM derived from enucleation. For validation, the results are compared with SNP array analysis, karyotyping, and Sanger sequencing of the *GNAQ* and *GNA11* genes.

## Material and Methods

### Tumour material

Archival frozen tumour samples of primary UM were obtained from 66 eyes containing UM that had been enucleated at the Leiden University Medical Center between 1999 and 2008. All tumours were lesions without prior treatment. Survival data were listed for use in this study (Table 1). Written informed consent was obtained for all patient samples. Tumour material was snap frozen using 2-methyl butane and DNA was isolated using the QIAmp DNA minikit (Qiagen, Valencia, USA) from 20 sections of 20μm according to the manufacturer’s guidelines.

**Table 1:** Tumour characteristics and survival data of 66 uveal melanoma patients.

Variable	Mean, median (range)	No. of patients (%)	Missing data (%)
**Age at diagnosis (years)**	60, 61 (12.8–88.5)		
**Male gender**		33 (50)	
**Largest tumor diameter (mm)**	13.5, 13 (8–30)		
**Tumor height (mm)**	7.7, 8 (1.5–12)		
**Cell type**			
-**Spindle**		21 (32)	
-**Epithelioid**		10 (15)	
-**Mixed**		35 (53)	
**Ciliary body involvement**		26 (41)	1 (1.6)
**Survival (months)**	59, 54 (2–157)		
**Survival status**			
-**Alive**		28 (42)	
-**Deaths due to metastasis**		34 (52)	
-**Deaths due to other cause**		4 (6)	
**TNM 7 stage**			
- **I-IIB**		43 (65)	
- **IIIA-IIIC**		23 (35)	

### Histopathology

Histologic sections were prepared from tissues fixed in 4% neutral-buffered formalin for 48 hours and embedded in paraffin. Hematoxylin-eosin–stained 4-μm sections were reviewed by one ocular pathologist for confirmation of the diagnosis and evaluated for histologic parameters, which included largest basal diameter (in millimeters), prominence (apical height, in millimeters), cell type according to the modified Callender classification, ciliary body involvement, and intrascleral in-growth [16].

### Karyotyping

Following enucleation, a small part of each tumor was sent out for cell culture. Following mechanical dissection of the tumor biopsy, cells were washed and placed into one flask with RPMI 1640 (15% fetal bovine serum [Invitrogen, Breda, The Netherlands]) medium and another flask with Amniochrome II (Cambrix Bio Science, Verviers, Belgium). The flasks were cultured at 37°C with 5% CO_2_ for up to 4 weeks and harvested when at least 75% of the surface was covered with cells (after a mean of 18 days; SD, 9.4 days). When cell culturing was successful, conventional karyotyping was performed, to determine the presence of chromosomal changes.

Two independent observers assessed all evaluations and scores, each without knowledge of the results obtained by the other investigator, to ensure accuracy of quantification of the slides. In case of a difference, consensus was reached during a simultaneous session.

Cytogenetic analysis was performed on GTG-banded (G-banding with trypsin and Giemsa) metaphases. In the case of a normal karyotype, at least 20 metaphases were analyzed. When an abnormal clone was detected in the first ten karyotyped cells, no further analysis was performed; when three cells with loss of 1 copy of chromosome 3 were observed, monosomy 3 was identified.

### Digital PCR (dPCR)

#### GNAQ/11 mutation detection

Presence of a mutation in either the *GNAQ* or *GNA11* gene was analysed using hydrolysis probes in a multiplex dPCR. Of each tumour sample 10ng of DNA was used in a 20ul reaction volume. The reaction mixture consisted of 2x droplet PCR supermix (Bio-Rad Laboratories, Inc.), 20x target probe (FAM), 20x wildtype probe (HEX). Proprietary probes and primers (Bio-Rad Laboratories, Inc.) were used and the sequence context is provided in S1 Table. Using a QX100 droplet generator and DG8 cartridges (Bio-Rad Laboratories, Inc.), each sample of 20ul was converted to an emulsion of 20.000 droplets. Emulsified samples were transferred to a 96-well PCR plate and the following protocol was used for PCR to end point using a T100 thermal cycler: 95°C, 10min; (94°C, 30sec; 55°C, 1min) 40x; 98°C, 10min; 4°C, till end. After PCR the plate was loaded into the QX100 droplet reader (Bio-Rad Laboratories, Inc.), each well was read serially. Digital PCR (dPCR) software (QuantaSoft) reads the positive and negative droplets in each sample and plots the fluorescence droplet by droplet. The positive droplets represent the concentration of the target allele in the samples. Digital PCR software allowed visualization of the data.

#### Copy Number Variation

Copy numbers of chromosome 3 and 8q were analysed using probes for *PPARG* and *PTK2* respectively. Because gain of 8q is often correlated with isochromosome formation, also a probe at 8p was analysed (*TUSC3*). In order to calculate normalized copy numbers, *TERT* (situated at chromosome 5) was used as reference. Thresholds for copy number analysis are: loss, <1.9: normal, 1.9–2.1: gain, >2.1-<3.1: amplification, >3.1.

Of each tumour sample 50–60ng of DNA was used in a 20ul reaction volume. The reaction mixture consisted of 2x droplet PCR supermix (Bio-Rad Laboratories, Inc.), 20x target probe (FAM), 20x reference probe (HEX). Sequence context is provided in S1 Table. Droplet generation, droplet reading and analysis were similar as in the mutation detection assay. The following end point PCR protocol was used: 95°C, 10min; (94°C, 30sec; 60°C, 1min) 40x; 98°C, 10min; 4°C, till end.

#### Sanger Sequencing

For validation of the *GNAQ* and *GNA11* mutation status, as acquired by dPCR, Sanger sequencing was performed on all 66 UM DNA samples by PCR using a Sybr green premixture from Bio-Rad Laboratories, Inc. Primers used are summarized in S2 Table, and the following PCR protocol was used for amplification of exon 4 and exon 5 of *GNAQ* and *GNA11* genes: 94°C, 3min; (96°C, 15sec; 63°C, 15sec; 72°C, 1min) 7x; (96°C, 15sec; 61°C, 15sec; 71°C, 1min) 8x; (96°C, 15sec; 60°C, 15sec; 72°C, 1min) 36x;72°C, 1min; till end. Following amplification DNA clean-up was performed using Nucleospin Extract II columns (Machery-Nagel, Düren, Germany) according to the manufacturer’s instruction. For Sanger sequencing analysis 10 pmol of the forward or reverse primer was added to the purified DNA amplicon. Sequencing for mutations was outsourced (Baseclear, Leiden, Netherlands). In UM samples showing no mutation in exon 5 of *GNAQ* or *GNA11* the exon 4 mutation status of both genes was determined (method identical to exon 5), primers are summarized in S2 Table. We used Mutation Surveyor software (Softgenetics, State College, USA) to assist mutation analysis.

### Single Nucleotide Polymorphism (SNP) analysis

We used SNP microarray data that was acquired for clinical purposes on UM samples to determine chromosomal aberrations. Two types of SNP microarray chips were used. The Affymetrix 250K_NSP, chip, which contains roughly 250 000 probes across the genome and the Affymetrix Cytoscan HD chip, with approximately 750 000 probes across the genome. A first set of 28 samples was analyzed with the Affymetrix 250K_NSP chip. Since this chip was no longer available, the remaining 36 samples were measured with the Affymetrix Cytoscan HD chip.

The analysis of the Affymetrix 250K_NSP chips was performed with the ‘Genotyping Console’ to determine the copy number values and the ‘GCT Browser’ to visualize the data (both from Affymetrix). The Affymetrix Cytoscan HD chips were analysed with ‘ChAS’ (Affymetrix). The chromosomal aberrations that were found for both chip versions were put in a database for further analysis.

### Statistical analysis

To compare survival between UM patients with chromosome 3 aberrations and chromosome 8q abnormalities we plotted Kaplan-Meier functions. Survival analysis was performed using the log-rank test. To compare individual groups we calculated Hazard ratios (HR) using Cox regression model. Pearson’s correlation test was used for correlation analysis of monosomy 3 and 8q copy number of SNP, dPCR and karyotype data. For statistical analysis we used SPSS V.20.0.1 (IBM SPSS Statistics, IBM Corporation, Armonk, New York, USA).

## Results

### *GNAQ* and *GNA11* mutation analysis

**Fig 1:**
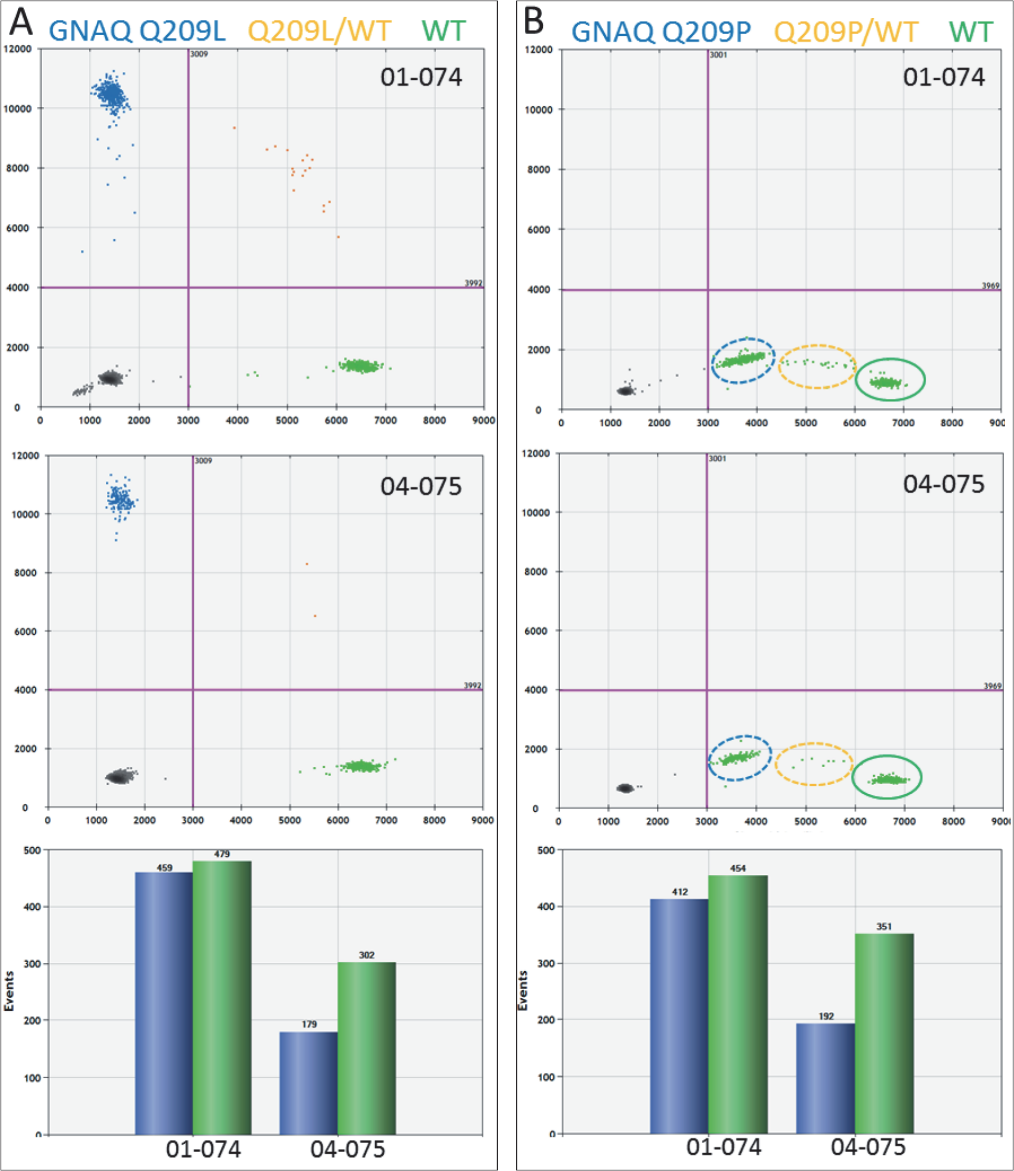
GNAQ mutation detection in UM by dPCR. Two color digital PCR is presented as a 2D plot. On the x-axis, the number of WT amplicons (green) is indicated and on the y-axis the mutant amplicons (blue) are counted. Empty PCRs and negative amplicons (black) end up in the bottom left quadrant. (A) PCRs containing WT and mutant (orange) will end up in the upper right quadrant. Based on Poisson distribution, the number of WT and mutant amplicons can be calculated (bar graph). This shows that 01–074 represent a homogeneous and 04–075 a heterogeneous UM sample. (B) Digital PCR analysis of these samples with the GNAQ Q209P assay did not result in mutant signals but aberrant signals on the x-axis. Manual selection of the aberrant signals in the WT quadrant of the plot learned that the clusters matched the positive clusters in the GNAQ Q209L assay. This indicates that the WT probe hybridized with low efficiency to the GNAQ Q209L allele in UM 01–074 and UM 04–075 in the absence of specific probe.

Using dPCR and mutation-specific probes, we analysed *GNAQ* and *GNA11* mutations in UM samples. Mutations were detected in both homogenous and heterogeneous samples. Assuming each UM cell contains a mutated and a wildtype allele of either *GNAQ* or *GNA11*, samples presenting equal numbers of mutant and wildtype alleles are considered homogenous. Hence, tumour samples that present an excess of wildtype alleles are considered heterogeneous. Fig. 1 shows two UM samples sharing the *GNAQ* Q209L mutation, caused by a substitution of an adenine for a thymine (c.626 A>T). Both samples tested positive for this mutation but the abundance of the mutation differs between these two samples. The allele distribution in UM 01–074 approaches a balanced mutant/wild type ratio (459/479) that fits a homogenous tumour (Fig. 1A). UM 04–075, on the other hand, clearly presents a mixed tumour with an excess of normal alleles (179 mutant/302 WT) (Fig. 1A). Both these tumours, UM 01–074 and UM 04–075, tested negative for the *GNAQ* Q209P mutation (Fig. 1B). However, in the absence of the *GNAQ* Q209L probe, the *GNAQ* wild type probe reacted with the *GNAQ* Q209L amplicon and thereby produced aberrant signals (Fig. 1B). The aberrant fractions with the *GNAQ* Q209P assay and the positive fractions with the *GNAQ* Q209L assay are similar in size and this supports the idea that the Q209L mutant allele in UM 01–074 and 04–075 gave rise to the aberrant fraction in the Q209P assay (Fig. 1A and 1B). This indicates that mutations can be detected in the absence of the specific probe for that mutation.

**Fig 2:**
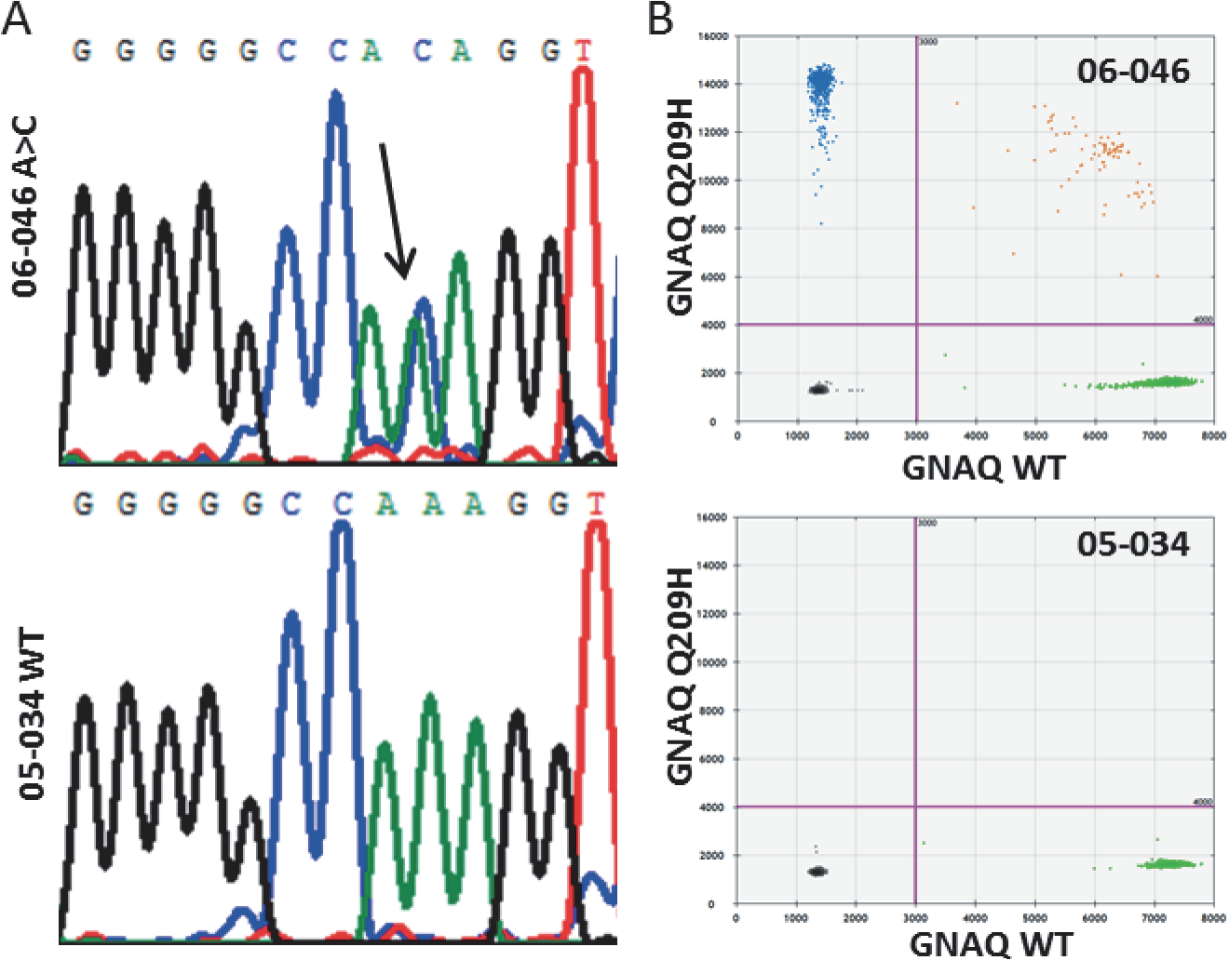
GNAQ A209H mutation in UM 06–046. (A) GNAQ mutation in 06–046 involves the c.627 A>C that encodes for the Q209H substitution. (B) For comparison, the WT sequence analysis of 05–034 is provided. Digital PCR with a newly designed GNAQ Q209H probe validates the mutation in 06–046.

Mutations in the GNAQ were observed in 27 UM, with 17 presenting the Q209P mutation and ten the Q209L mutation. Mutations in *GNA11* were more common as 33 UM tested positive for the *GNA11* Q209L mutation (Table 2). Only six out of 66 UM displayed a wildtype *GNAQ* and *GNA11* initially. However, rare mutations were detected in *GNAQ* and *GNA11* due to a minor cross reactivity of the probes, similar to what we observed in the *GNAQ* Q209P assay with *GNAQ* Q209L mutant alleles (Fig. 1B). UM 06–046 showed no positive signals with either of the *GNAQ* mutant probes but an aberrant fraction in both *GNAQ* assays indicated the presence of another mutation. Sanger sequencing revealed the c.627 A>C mutation that encodes for the *GNAQ* Q209H mutant (Fig. 2A). A newly developed dPCR probe for this mutation confirmed the presence of this mutation in UM 06–046 (Fig. 2B). Validation of an aberrant amplicon in *GNA11* of UM 02–167 revealed a double mutation (c.626_627 AG>TC). This mutation encodes also for the *GNA11* Q209L mutant because the additional base substitution does not alter the coding capacity. Since the second substitution is contained within the recognition sequence of the probe for the *GNA11* Q209L mutation, it interferes with accumulation of a positive signal in dPCR. Direct detection with dPCR and indirect detection of mutants combined, we detected exon 5 *GNAQ* and *GNA11* mutations in 94% of the UM. All mutations have been confirmed by sequence analysis, though mutant sequence signals in heterogeneous samples with an excess of wildtype DNA could be very low. In 4 out of the 62 cases with mutations detected with dPCR, sequence analysis only showed a minor mutant signal that on itself would be insufficient to call a tumour mutant. The remaining 4 UM that do not present mutations in exon 5 were analysed for exon 4 mutations of *GNAQ* and *GNA11*. This revealed a mutation at codon 183 (c.548G>A) of GNAQ in UM 08–004. In a total of 66 UM, 63 carried mutations in *GNAQ* and *GNA11*.

**Table 2:** Summary of GNAQ and GNA11 mutations detected in 66 UM samples.

	GNAQ				GNA11	Wildtype
	R183Q	Q209P	Q209L	Q209H	Q209L	-
**No. of tumours**	1	17	10	1	33+1*	3
% **of tumours**	1.5	25.8	15.1	1.5	51.5	4.6

* *GNA11* Q209L (c.626_627 AG>TC)

### Monosomy 3 and 8q gain

**Fig 3:**
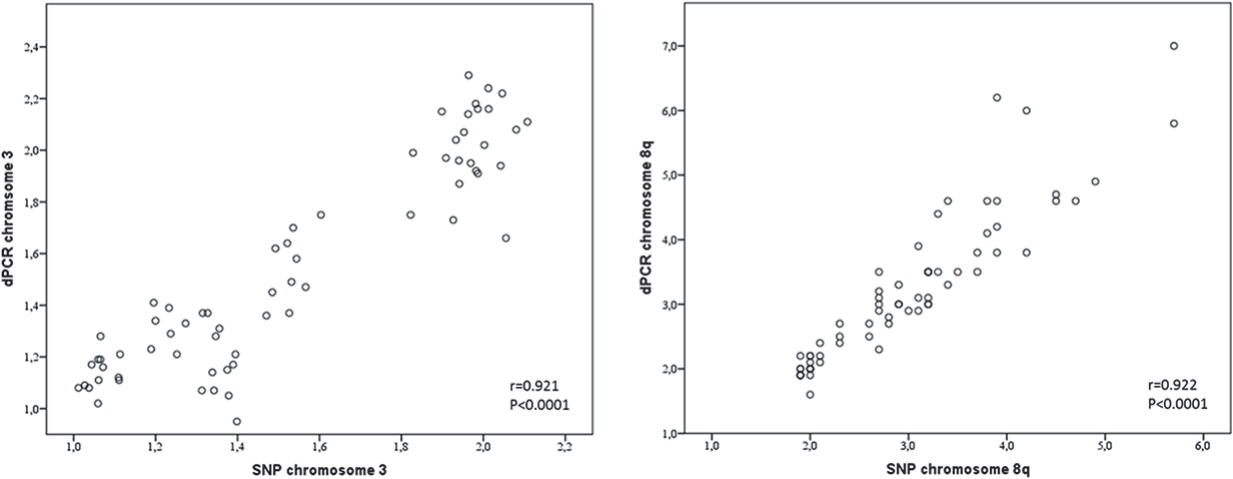
Correlation between dPCR and SNP with regard to chromosome 3 and 8q copy numbers. Copy numbers of chromosome 3 and 8q in UM calculated with SNP and dPCR display a strong correlation (r = 0.921 and r = 0.922, p<0.0001), indicating that these methods cross validate each other.

With dPCR we studied chromosome 3 and chromosome 8 status in UM. Using probes for *PPARG* at chromosome 3p, *PTK2* at chromosome 8q, and *TUSC3* at 8p, monosomy 3 and chromosome 8 abnormalities were analysed in UM (Table 3). Genomic aberrations detected with dPCR were validated by SNP array analysis and significant correlations (p≤0.01) between SNP array and dPCR analysis for monosomy 3 (r = 0.921) and 8q gain (r = 0.922) were observed (Fig. 3). Comparison with karyotyping revealed that in almost a quarter of the cases (n = 15), karyotyping was not successfully applied. Monosomy 3 or 8q aberrations were not detected in 13 and 17 of the cases, respectively, where SNP array and dPCR did detect them. In one case (02–199) monosomy 3 was detected by karyotyping but was not detected by SNP array and dPCR (Table 3). However, still significant correlations were observed for both chromosome 3 and 8 (r = 0.528, p = 0.0002; r = 0.455, p = 0.001) when comparing dPCR and karyotyping.

**Table 3:** Monosomy 3 and chromosome 8 copy number variation: SNP array versus dPCR analysis.

	chromosome 3			Chromosome 8q		Chromosome 8p		Chrom 8
Tumour ID	SNP	dPCR	karyotype	SNP	dPCR	SNP	dPCR	karyotype
99–184	1.5	1.4	0	3.3	4.4	2.5	2.5	0
99–187	2.0	2.2	0	2.0	2.2	1.9	2.4	0
99–239	1.4	0.9	1	3.3	3.5	2.3	1.9	2
20–005	1.4	1.2	0	2.9	3.3	1.3	1.1	0
20–042	1.5	1.6	1	3.1	3.9	1.8	1.7	2
20–125	2.0	2.2	0	2.7	3.2	1.9	1.9	0
20–128	1.5	1.5	1	3.9	6.2	2.0	2.0	2
20–173	1.3	1.3	1	2.3	2.5	1.7	1.6	2
20–178	1.3	1.1	0	4.2	6.0	2.1	2.2	0
01–042	2.0	2.1	0	1.9	1.9	2.0	2.0	0
01–074	1.9	2.2	0	2.7	3.5	2.3	2.4	0
01–091	1.4	1.2	0	3.8	4.6	2.1	2.0	0
01–129	1.8	1.8	n.a.	2.1	2.2	2.2	2.1	n.a.
01–131	1.5	1.5	n.a.	3.4	4.6	1.4	1.3	n.a.
02–158	2.0	2.3	0	2.0	2.2	2.0	2.2	0
02–167	1.6	1.5	n.a.	2.1	2.1	2.0	2.0	n.a.
02–174	1.4	1.1	0	2.6	2.7	2.7	2.8	0
02–189	1.4	1.2	1	2.3	2.4	1.8	1.6	2
02–199	1.8	2.0	1	1.9	2.0	2.0	2.2	0
03–031	1.4	1.3	1	3.9	4.6	1.4	1.3	2
03–086	2.0	2.2	0	1.9	2.0	2.1	2.2	0
03–087	2.1	1,7*	0	2.0	1,6*	2.0	1,6*	0
03–120	2.0	2.0	n.a.	1.9	1.9	1.9	2.0	n.a.
03–129	1.5	1.4	0	2.7	3.1	1.9	2.1	0
04–018	1.9	2.0	0	2.3	2.7	2.4	2.6	0
04–035	1.3	1.1	1	2.7	3.0	1.2	1.0	2
04–074	2.0	1.9	n.a.	2.0	2.0	2.0	1.9	n.a.
04–075	1.9	2.0	0	2.9	3.0	1.9	2.1	0
04–103	2.0	1.9	0	2.0	1.9	2.1	2.0	0
04–112	1.3	1.1	1	3.5	3.5	2.0	1.8	2
05–005	1.3	1.4	0	3.2	3.1	2.0	1.8	0
05–020	1.9	1.9	n.a.	4.5	4.6	2.0	1.8	n.a.
05–033	1.1	1.2	1	2.1	2.4	1.9	1.9	2
05–034	1.5	1.6	1	3.1	3.1	3.0	2.8	1
05–046	1.1	1.1	1	3.7	3.5	1.9	1.8	2
05–058	2.0	1.9	0	1.9	1.9	2.1	1.8	0
05–061	1.0	1.1	n.a.	5.7	5.8	3.4	3.5	n.a.
06–004	1.1	1.1	n.a.	4.2	3.8	1.1	1.0	n.a.
06–008	1.2	1.4	1	2.9	3.0	2.0	2.0	0
06–009	1.1	1.0	1	3.2	3.0	2.0	3.0	1
06–010	2.0	2.0	n.a.	2.0	2.0	1.3	1.2	n.a.
06–011	2.0	2.1	n.a.	3.2	3.5	2.5	2.3	n.a.
06–014	1.6	1.8	0	4.5	4.7	1.6	1.7	1
06–015	1.0	1.2	1	3.9	3.8	1.3	1.3	2
06–023	1.3	1.4	1	2.6	2.5	2.3	2.0	2
06–033	2.1	2.1	0	3.2	3.0	2.8	2.8	1
06–036	2.0	2.2	0	3.1	2.9	2.9	2.9	1
06–038	1.1	1.1	0	3.4	3.3	1.2	1.1	0
06–041	1.2	1.3	1	4.7	4.6	1.3	1.1	2
06–042	1.1	1.3	1	3.0	2.9	1.0	1.1	2
06–045	1.5	1.6	n.a.	3.8	4.1	1.5	1.4	n.a.
06–046	1.9	1,7*	n.a.	2.7	2,3*	2.0	1,6*	n.a.
06–047	1.2	1.4	0	2.8	2.7	1.3	1.2	0
07–003	2.0	2.2	n.a.	1.9	2.2	2.1	2.1	n.a.
07–004	2.1	2.1	0	2.0	2.0	2.0	2.0	0
07–005	1.3	1.3	1	3.9	4.2	1.2	1.2	2
07–007	1.0	1.1	0	1.9	1.9	2.0	2.1	0
07–012	1.5	1.7	n.a.	3.7	3.8	2.0	1.9	n.a.
07–030	1.1	1.2	1	3.2	3.5	1.3	1.3	2
07–034	1.9	2.0	0	2.0	2.1	2.1	2.1	0
07–047	1.0	1.1	1	2.0	2.0	2.1	1.9	0
07–050	1.1	1.2	1	4.9	4.9	2.9	3.1	2
08–004	1.2	1.2	n.a.	2.8	2.8	2.1	1.9	n.a.
08–005	1.3	1.2	1	5.7	7.0	1.8	1.8	2
08–008	1.1	1.2	0	2.7	2.9	2.0	2.1	0
08–029	1.2	1.3	0	3.2	3.5	2.1	2.1	0

SNP and dPCR thresholds: loss, <1.9: normal, 1.9–2.1: gain, >2.1- <3.2: amplification, >3.1

* Aberration at chromosome 5, location of TERT, no proper correction possible with TERT dPCR Karyotype chromosome 3: 0; Disomy, 1; MonosomyKaryotype chromosome 8: 0; Disomy, 1; gain, 2; isochromosomen.a.: not analyzed

**Fig 4:**
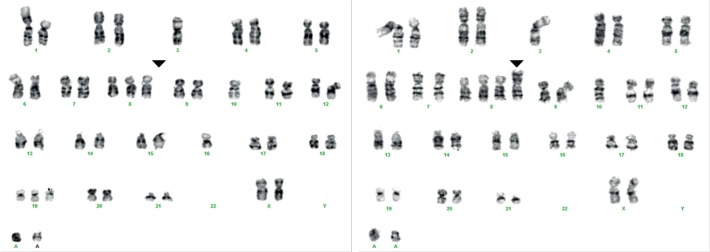
Chromosome 8 heterogeneity in UM 07–050. One clone presents trisomy 8 (left) while the other clone presents trisomy 8 (triangle) in combination with isochromosome 8q (right). Besides chromosome 8, chromosome 1, 3, 10 and 22 showed clonal abnormalities. Two markers (A) on the bottom indicate the shared origin of these clones.

In 66 UM, 52 displayed a gain of 8q (79%) while 43 UM showed monosomy 3 (65%). Based on chromosome 3 analysis, UM can be divided in 2 groups, while UM is divided into 3 classes based on 8q copy number. Fourteen tumours displayed a normal 8q copy number(1.8<n<2.2), 24 UM displayed a 8q gain of one copy (2.1<n<3.2), while 28 UM presented a 8q gain of more than 1 copy which we categorised as amplification of 8q (n>3.1). A balanced increase of 8q and 8p copy number (Table 3) furthermore indicated that 8q gain is in 8/24 cases (99–187, 02–158, 02–174, 04–018, 05–034, 06–009, 06–033, 06–036) due to trisomy 8. In the remainder (16/24) of the UM with 8q gain, an excess gain of 8q compared to 8p indicated 8q isochromosome formation. Amplification of 8q coincided with 8q/8p imbalance and isochromosome formation. However, in six cases with 8q/8p imbalance a gain of 8p was also observed (99–184, 20–178, 01–074, 05–061, 06–011 and 07–050), suggesting trisomy 8 in combination with 8q isochromosome formation. With cytogenetic analysis, gain of chromosome 8 in combination with 8q isochromosome was confirmed in UM 07–050 (Fig. 4).

**Fig 5:**
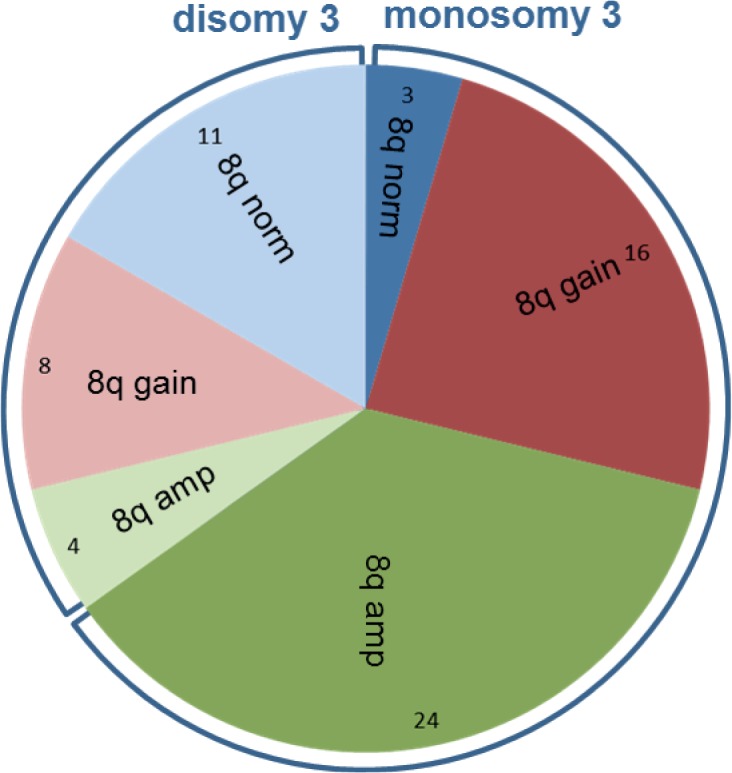
Non-random distribution of monosomy 3 and chromosome 8 aberrations. In 40 (61%) of the 66 UM monosomy 3 and 8q aberrations occur together. Non-random distribution is even stronger for 8q amplification and monosomy 3, as monosomy 3 is present in 24 (86%) of 28 UM with 8q amplification.

Monosomy 3 and 8q aberrations mostly occurred together (n = 40) (p<0.01) (Fig. 5). In total, 3 UM with monosomy 3 and a normal chromosome 8 karyotype were detected while 12 UM with chromosome 8 aberrations were detected (8 gains and 4 amplifications) in the disomy 3 group.

**Fig 6:**
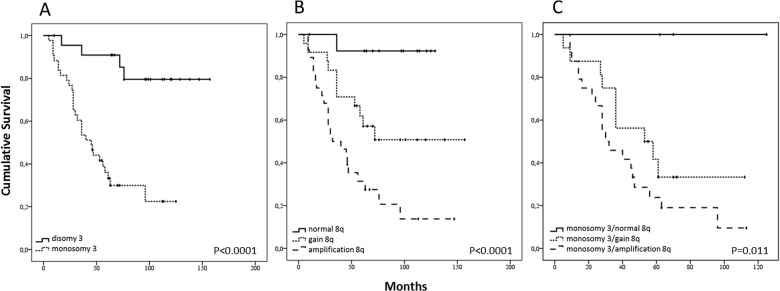
Worst survival for patients with amplification of chromosome 8q. The presence of monosomy 3 (n = 43) in UM is highly prognostic for death due to metastases compared to the presence of disomy 3 (n = 23) (p<0.0001). (A) Significant differences in survival between classes of tumours with normal (n = 14), gain (n = 24), or amplification (n = 28) of chromosome 8q. A significant difference in survival was observed between the three categories (p<0.0001), and between the different individual classes: normal vs. amplification (p<0.0001), and gain vs. amplification (p = 0.00125). Between normal and gain of 8q a trend towards significance was observed (p = 0.07). (B) On the background of monosomy 3, 8q amplification (n = 24) increased the risk significantly (p = 0.011) compared to monosomy 3 with 8q gain (n = 16). Three UM presented monosomy 3 without aberration on 8q: none died due to UM metastasis. (C)

Monosomy 3 is highly prognostic for death due to metastasis, and 5-year survival is 37% in this group compared to 90% 5-year survival in the UM expressing disomy 3 (Fig. 6A). Survival analysis showed that amplification of 8q (n = 28) is associated with a bad prognosis and only 29% of these patients survive past 5 years (Fig. 6B). UM with normal copy numbers of 8q (n = 14) displayed a good prognosis with a 5-year survival of 93%. UM with 8q gain (n = 24) presented an intermediate prognosis and a 5-year survival of 67% (Fig. 6B). To investigate whether monosomy 3 and 8q risk are additive, we evaluated survival in UM presenting monosomy 3 in combination with gain or amplification of 8q. The survival of UM patients is significantly (p 0.011) worse if monosomy 3 is combined with 8q amplification (Fig. 6C). The 5-year survival drops from 44% to 25% in patients with a UM that present monosomy 3 in combination with 8q amplification compared to UM presenting monosomy 3 in combination with 8q gain. Monosomy 3 without chromosome 8 aberration was observed in only 3 patients and metastasis was not detected in these patients (Figs. 5 and 6C).

## Discussion

With six digital PCR runs, we were able to reconstitute the genomic profile of 66 UM. Using three validated assays, we analysed *GNAQ/GNA11* recurrent hotspot mutations. Two samples presented rare mutations in *GNAQ* (c.627A>C) and *GNA11* (c.626_627 AG>TC) which resulted in false-negative results in dPCR. However, both variants presented aberrant signals in the *GNAQ* and *GNA11* assays that indicated the presence of rare variants. Whether all possible rare variants in *GNAQ* and *GNA11* will result in aberrant signals when analysed with the standard assays is not certain but in this panel of 66 UM, no additional variants were detected with sequence analysis. Direct and indirect mutation detection with dPCR revealed *GNAQ/GNA11* mutations in 94% of the UM. This is higher than the mutation frequencies that have been reported and this is at least in part explained by the sensitivity of the dPCR in heterogeneous UM [2,3].

Overall, a good correlation existed between dPCR analysis and copy number analysis with SNP arrays (Fig. 3). However, in two UM, genomic profiling with dPCR turned out to be incorrect because SNP analysis revealed a gain in the genomic region containing the reference gene (TERT). Using multiple reference genes from stable regions in dPCR in a multi-colour approach would solve the problem with *TERT* normalisation. UM are relatively stable and normalisation of 3 or 4 reference genes will be sufficient to identify aberrant reference genes that should be excluded from analysis. These findings indicate that dPCR and SNP arrays are both valid means to quantify gene copy number. Since dPCR is time and cost effective, it would make it the method of choice. Application of quantitative copy number analysis furthermore improves diagnosis and prognosis. Quantitative analysis of 8q copy number validated the dosage effect of 8q on prognosis that was previously shown but which has not yet been widely implemented in UM prognostication [4,6]. Gain of one copy of 8q (2.1<n<3.2) is correlated with a moderate risk of metastasis while higher gains (amplification, n>3.1) are correlated with an even worse prognosis. Amplifications are correlated with isochromosome 8q formation which we validated with imbalance of 8q/8p copy numbers in dPCR. Isochromosome formation also occurred in combination with trisomy 8 in 6 UM. Coexistence of clones with isochromosome 8q and trisomy 8 has been interpreted as the consequence of independent events [17]. In an experimental model however, isochromosome formation was shown to be secondary to gain of a chromosome [18]. We therefore propose that isochromosome 8q formation could be secondary to trisomy 8 and frequent detection of heterogeneous UM that present both trisomy 8 and isochromosome 8q support this progression model (Table 3, Fig. 4) [9]. Recently, chromosome 8p loss was identified as independent risk predictor of poor outcome [11]. As isochromosome 8q formation in UM is often associated with 8p loss, we propose that isochromosome formation may be an underlying cause. Superimposing monosomy 3 in this progression model furthermore suggests that increasing 8q copy numbers due to isochromosome 8q formation is accompanied by monosomy 3 formation. Just like the underrepresentation of monosomy 3 UM with normal chromosome 8, UM with 8q amplification and a normal chromosome 3 are virtually lacking (Fig. 5). Chromosome 8 aberrations are also most common in other studies and this supports that chromosome 8 aberrations proceed monosomy 3 [7,11,19–21].

Regardless of the order of events, a combination of monosomy 3 and chromosome 8 gain is correlated with a worse prognosis than monosomy 3 on itself [7]. Moreover, an increased risk of metastasis formation in the presence of 8q amplification in combination with monosomy 3 compared to monosomy 3 in combination with gain of 8q supports a dominant role for 8q dosage in UM metastases (Fig. 6C) [4]. We therefore propose that molecular prognostication of UM should include 8q quantification. With quantitative SNP analysis, 8q copy number can be adequately determined while dPCR provides a cost and time effective alternative. Moreover, the sensitivity of dPCR specifically facilitates the analysis of mutations and copy number aberrations in small and highly diluted samples such as circulating tumour cells and free circulating tumour DNA. Future implementation of quantification of 8q in prediction models will improve prognostication in UM.

## Supporting Information

S1 Table dPCR sequences context. (XLSX)

S2 Table GNAQ and GNA11 primers used for Sanger sequencing. (XLSX)

S3 Table Hazard ratios chromosome 8q. (XLSX)
